# Buruli ulcer: An epidemiological update from Japan

**DOI:** 10.1111/1346-8138.17483

**Published:** 2024-09-30

**Authors:** Ryo Fukaura, Manabu Ato, Chiaki Murase, Yuji Miyamoto, Mariko Sugawara‐Mikami, Toshifumi Takahashi, Yoshihiko Hoshino, Noriki Fujimoto, Masashi Akiyama, Norihisa Ishii, Rie Yotsu

**Affiliations:** ^1^ Department of Dermatology Nagoya University Graduate School of Medicine Nagoya Japan; ^2^ Leprosy Research Center National Institute of Infectious Diseases Tokyo Japan; ^3^ Sugawara Dermatology Clinic Yokohama Japan; ^4^ Department of Dermatology Shiga University of Medical Science Otsu Japan; ^5^ National Sanatorium Tama Zenshoen Tokyo Japan; ^6^ Department of Tropical Medicine and Infectious Disease Tulane School of Public Health and Tropical Medicine New Orleans Louisiana USA; ^7^ Department of Dermatology National Center for Global Health and Medicine Tokyo Japan; ^8^ School of Tropical Medicine and Global Health, Nagasaki University Nagasaki Japan

**Keywords:** Japanese, *mycobacterium ulcerans* subsp. *shinshuense*, mycolactone, neglected tropical disease, transmission

## Abstract

Japan is one of the rare non‐tropical countries with documented cases of Buruli ulcer (BU). *Mycobacterium ulcerans* subsp. *shinshuense* has been identified as the causative agent. The first report of BU in Japan dates back to 1982, with sporadic reports thereafter. Recently, the number of cases has been on the increase, and 50 cases (57.7%) are from the past decade alone, out of a total of 87 cases reported to date. Japan's well‐developed healthcare facilities play a crucial role in enabling detailed investigations and providing appropriate treatment for patients, contributing to a favorable prognosis. However, the rarity of the disease results in lack of awareness among healthcare professionals, leading to frequent delays in diagnosis. This article aims to offer an updated overview of BU cases in Japan and to raise awareness of BU among dermatologists and other healthcare professionals in a non‐endemic setting.

## 1 INTRODUCTION

Buruli ulcer (BU) is a non‐tuberculous mycobacterial (NTM) infection caused by *Mycobacterium (M.) ulcerans*. Patients present with large, necrotizing ulcers that can develop on any part of the body, but often on the exposed sites. If not diagnosed and treated early, it may lead to permanent disfiguration and disabilities.

BU has been designated as a ‘skin neglected tropical disease (skin NTD)’ by the World Health Organization (WHO), alongside conditions such as lymphatic filariasis, cutaneous leishmaniasis, and mycetoma.^1^ Epidemiological data of BU for routinely collected by the WHO. It has been reported from over 33 countries, with 14 countries reporting to WHO on a regular basis. The majority of them are found in tropical and subtropical regions, especially in sub‐Saharan Africa.^2^, ^3^ However, cases have also been reported from Japan and Australia, which are outside of these climate zones.

The report of the first case of BU in Japan was published in 1982.^4^ To date, a total of 87 cases have been diagnosed from the country (55 female, 32 male cases), and it has been established that the causative organism for cases within Japan is *Mycobacterium ulcerans* subsp. *shinshuense* (*M. shinshuense*). We have published our case summaries previously.^5^, ^6^, ^7^, ^8^ In this article, we aim to provide an update of the epidemiology of BU in Japan, with a focus on the characteristics of the cases diagnosed in the past 6 years, from January 2017 to December 2022.

## 2 METHODS

The last report on BU status in Japan was published by Suzuki et al.^8^ and detailed 60 cases identified in Japan by the end of 2016. Since then, 27 additional cases have been reported between January 2017 and December 2022. These new cases were identified after their respective clinical teams reported the diagnosis to the National Institute of Infectious Diseases (NIID). Following notification, each team was contacted to obtain further relevant information, including site, size, category of the lesions, patient location, treatments, and outcomes. These details are summarized in Table 1. Several of these cases have already been reported individually.^9^, ^10^, ^11^, ^12^ This study was reviewed and approved by the medical research ethics committee of the NIID for inclusion of human subjects (#1369). All research practices were performed in accordance with the Declaration of Helsinki.

**TABLE 1:** Summary of BU cases diagnosed in Japan, 2017–2022.

Year	Age	Sex	Comorbidities	Prefecture	Month of symptom onset	Water source	Site of lesion	Max diameter	Category	Causative organism	Antibiotics	Duration	Surgery	Outcome
2017	87	M	Unknown	Hyogo	Jan	No	Left elbow	3.5 cm	I	*M. shinshuense*	RFP, CAM, LVFX	Unknown	Pocket incision	Cured
2017	66	F	Unknown	Tottori	Dec	No	Left leg	4 cm	I	*M. shinshuense*	RFP, CAM, LVFX	4 months	Skin graft	Cured
2017^9^	4	F	Unknown	Iwate	Oct	No	Left arm	14.5 cm	II	*M. shinshuense*	RFP, CAM, TSFX	Unknown	Skin graft	Cured
2017	87	F	Unknown	Nagano	Jan	No	Right leg	4 cm	I	*M. shinshuense*	RFP, CAM, LVFX	1 month	Skin graft	Died from unrelated causes
2017	63	M	Unknown	Shiga	Mar	Yes	Left leg	3 cm	I	*M. shinshuense*	RFP, CAM, LVFX	2 months	None	Cured
2017	88	F	Unknown	Mie	Jan	No	Left hand	6 cm	II	*M. shinshuense*	RFP, MNO, LVFX	4 months	None	Cured
2017	76	M	Unknown	Hyogo	Mar	No	Right arm	4 cm	I	*M. shinshuense*	RFP, CAM, LVFX	2 months	Debridement	Cured
2018	5	F	Unknown	Mie	Nov	No	Left leg	2 cm	I	*M. shinshuense*	RFP, CAM, TSFX	Unknown	None	Cured
2018	73	M	Unknown	Nagano	Jan	No	Left hand	2.7 cm	I	*M. shinshuense*	Unknown	Unknown	Debridement	Cured
2018	40	M	Unknown	Wakayama	Unknown	Yes	Left arm	1 cm	I	*M. shinshuense*	RFP, CAM, LVFX	3 months	None	Cured
2019	47	M	Unknown	Kyoto	Feb	No	Right arm	2 cm	I	*M. shinshuense*	RFP, CAM, LVFX	Unknown	Skin graft	Cured
2019^10^	29	M	Unknown	Hyogo	Oct	Yes	Right leg	2.5 cm	I	*M. shinshuense*	RFP, CAM, MXFX	Unknown	Skin graft	Cured with permanent joint contraction
2019^11^	70	F	Unknown	Okayama	Oct	No	Left hand	4 cm	II	*M. shinshuense*	CAM, LVFX	8 months	None	Cured
2020	38	F	Unknown	Shizuoka	Nov	No	Right arm Left leg	1 cm, 3 cm	III	*M. shinshuense*	RFP, CAM, MXFX	Unknown	None	Cured
2020	39	M	Unknown	Miyagi	Oct	Yes	Left leg	1.5 cm	I	*M. shinshuense*	RXM, LVFX	6 weeks	Skin graft	Cured
2020	89	F	Unknown	Akita	Jun	Yes	Left leg (5 ulcers)	4 cm, 2 cm, 5 mm	III	*M. shinshuense*	RFP, CAM, LVFX	Unknown	None	Cured
2020	70	F	Type 2 diabetes, Hypertension	Aichi	Jan	No	Left face Left neck	5.5 cm	III	Unknown	RFP, CAM	2 months	Ear amputation	Cured
2020	52	F	Bronchogenic cyst	Shiga	Nov	No	Left arm	3 cm	I	Unknown	None (Surgery only)	None	Excision with 6 mm margin	Cured
2021	58	F	None	Nagano	Oct	No	Left arm	1.2 cm	I	*M. shinshuense*	RFP, CAM, LVFX	14 weeks	Debridement	Cured
2021	73	M	Hypertension, hypercholesterolaemia	Hyogo	May	No	Right leg	2 cm	I	Unknown	RFP, CAM, LVFX	11 weeks	Excision	Cured
2021	79	F	Hypercholesterolaemia, osteoporosis	Shiga	Dec	No	Left arm	4 cm	I	Unknown	RFP, CAM, LVFX	8 weeks	None	Cured with scarring
2021	20	F	Deep mycoses of the skin	Shiga	Jul	Yes	Left face	1.5 cm	III	Unknown	RFP, CAM, LVFX	5 weeks	None	Cured with scarring
2022	49	F	Marfan's syndrome	Tottori	Jan	No	Left arm	3 cm	I	Unknown	RFP, CAM	8 weeks	Skin graft	Cured
2022	80	M	Chronic kidney disease, atrial fibrillation, hypertension, stroke lumbar stenosis	Nagano	Feb	No	Right face	20 cm	III	*M. shinshuense*	RFP, CAM, LVFX	6 weeks	Skin graft	Cured
2022	7	F	None	Toyama	Nov	Yes	Left arm	2 cm	I	*M. shinshuense*	RFP, CAM, TSFX	Unknown	Skin graft	Cured with scarring
2023	83	F	Unknown	Tochigi	Dec	Unknown	Unknown	Unknown	Unknown	Unknown	Unknown	Unknown	Unknown	Unknown
2023^12^	77	F	Ulcerative colitis	Aichi	Nov	No	Left leg	7 cm	II	*M. shinshuense*	RFP, CAM, LVFX	3 months	Skin graft	Cured with scarring

Abbreviations: BU, Buruli ulcer; CAM, clarithromycin; LVFX, levofloxacin; MNO, minocycline; *M*. *shinshuense*, *Mycobacterium ulcerans* subsp. *shinshuense*; MXFX, moxifloxacin; RFP, rifampicin; RXM, roxithromycin; TSFX, tosufloxacin.

## 3 RESULTS

**FIGURE 1:**
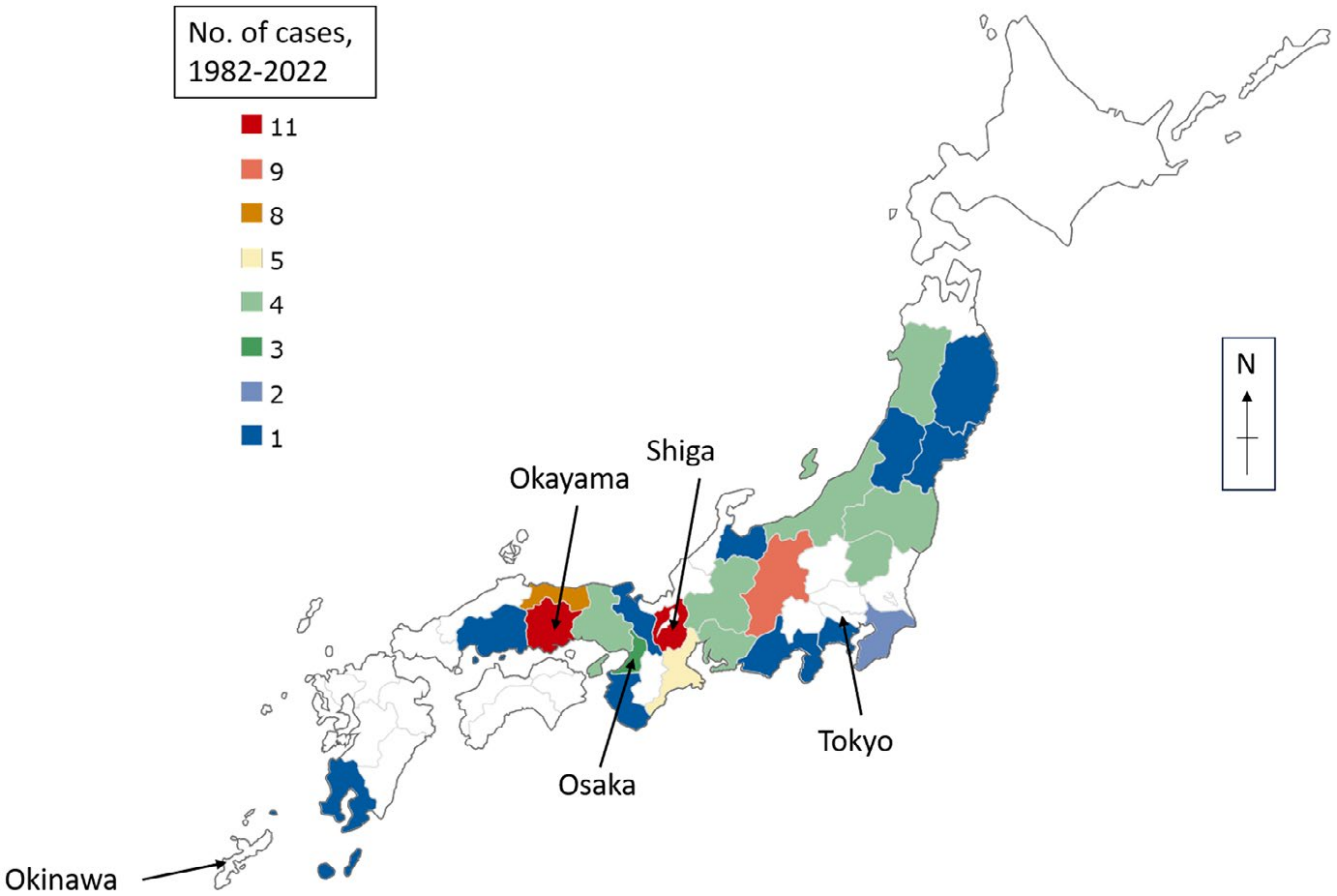
Map of Japan showing the prefectures from where BU cases have been reported so far.

The geographical pattern of BU cases in Japan is shown in Figure 1. They are sporadically distributed across the island of Honshu, encompassing both metropolitan and rural areas. Certain regions, such as Okayama and Shiga prefectures, exhibit ‘hot‐spot’‐like concentrations of cases; however, further accumulation of cases is necessary before any significant associations can be drawn. Interestingly, despite BU being typically considered a ‘tropical’ disease, there have been no reports from the southern islands of Okinawa and only one from the Kyushu area (none in the most recent set of reported cases). These regions have climates closer to tropical countries than the prefectures of Honshu. It remains unclear whether BU is truly non‐existent in these areas, or if cases are undiagnosed or misdiagnosed. Further research is warranted to assess BU prevalence in these regions.

The most frequent presentation in the recent cohort of cases (2017–2022) was a small ulcer detected on a limb, which was refractory to treatment and exhibited rapid growth. The majority of lesions appeared on the limbs (92.6%, 25 out of 27 cases), while three patients (11.1%) presented with lesions on the face. Two patients presented with multiple ulcers.

In terms of diagnosis, polymerase chain reaction (PCR) for *M. ulcerans* subsp. *shinshuense* was positive in all 20 cases for which information was available. Of the seven cases that lacked 16s rRNA results, at least four cases had positive IS*2404* PCR results. The status of the remaining cases is unclear as data have not been made available. BU is not strictly a reportable disease in Japan, and although efforts were made to contact the attending clinical teams to request data, this has not always been successful. Among the 19 cases where mycobacterial culture was performed, seven (36.8%) were positive. An additional 12 cases (63.2%) had recorded negative cultures for mycobacteria, with cultures only growing bacteria of the normal skin flora, such as *Staphylococcus* or *Serratia* species. ZN staining was performed in three cases, all of which were positive. The diagnosis was made comprehensively based on these results. In two cases, all three diagnostic tests were positive.

**FIGURE 2:**
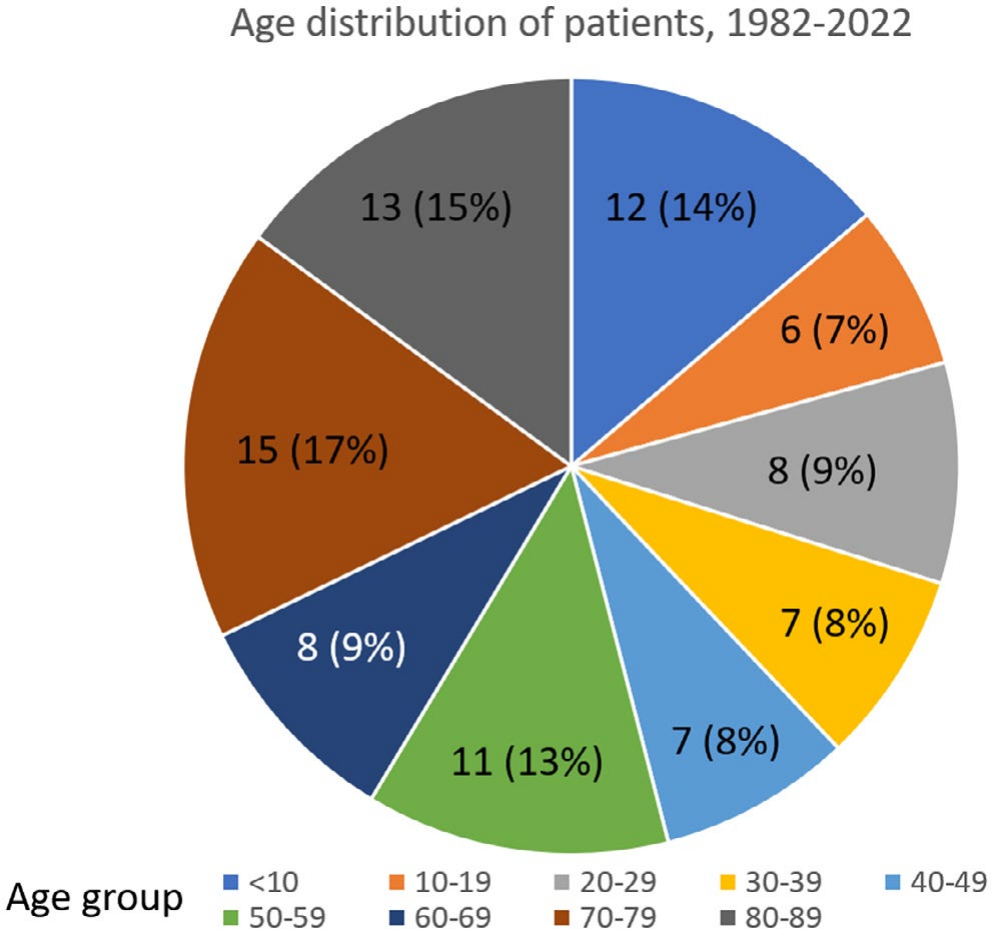
Age distribution of all reported cases of BU.

It has been well‐established that BU affects patients of all age groups in Japan.^6^ This pattern continued in the most recent cohort (2017–2022), with the patient ages ranging from 4 to 89 years old. Within these cases, 55.6% (15 out of 27 cases) were diagnosed in people over the age of 60. When examining the overall statistics from 1982 onwards, 41.4% (36 of 87 cases) were diagnosed in people over the age of 60 (Figure 2). The apparent concentration of cases among the elderly may primarily reflect Japan's aging population pyramid rather than specific pathogen behavior, differing from trends observed in most other endemic countries.^13^ Australia, which is also faced with an aging population, shows similar age distributions of BU to Japan, with approximately 40% of cases occurring in the elderly.^14^

Suzuki et al.^8^ previously reported an association between BU development and water exposure in Japan, along with a strong seasonal correlation. In the most recent cohort of cases (2017–2022), seven patients (out of 27, 25.9%) had a history of close contact with a water source. This includes regular occupational or leisure contact (e.g., farming or gardening), living in close proximity to a water source (e.g., rivers or lakes), or a recent history of significant water exposure (e.g., swimming or other watersports, or natural disasters). Overall, 20.7% of all cases (18 out of 87 cases, 1982 onwards) had confirmed close water contact.

**FIGURE 3:**
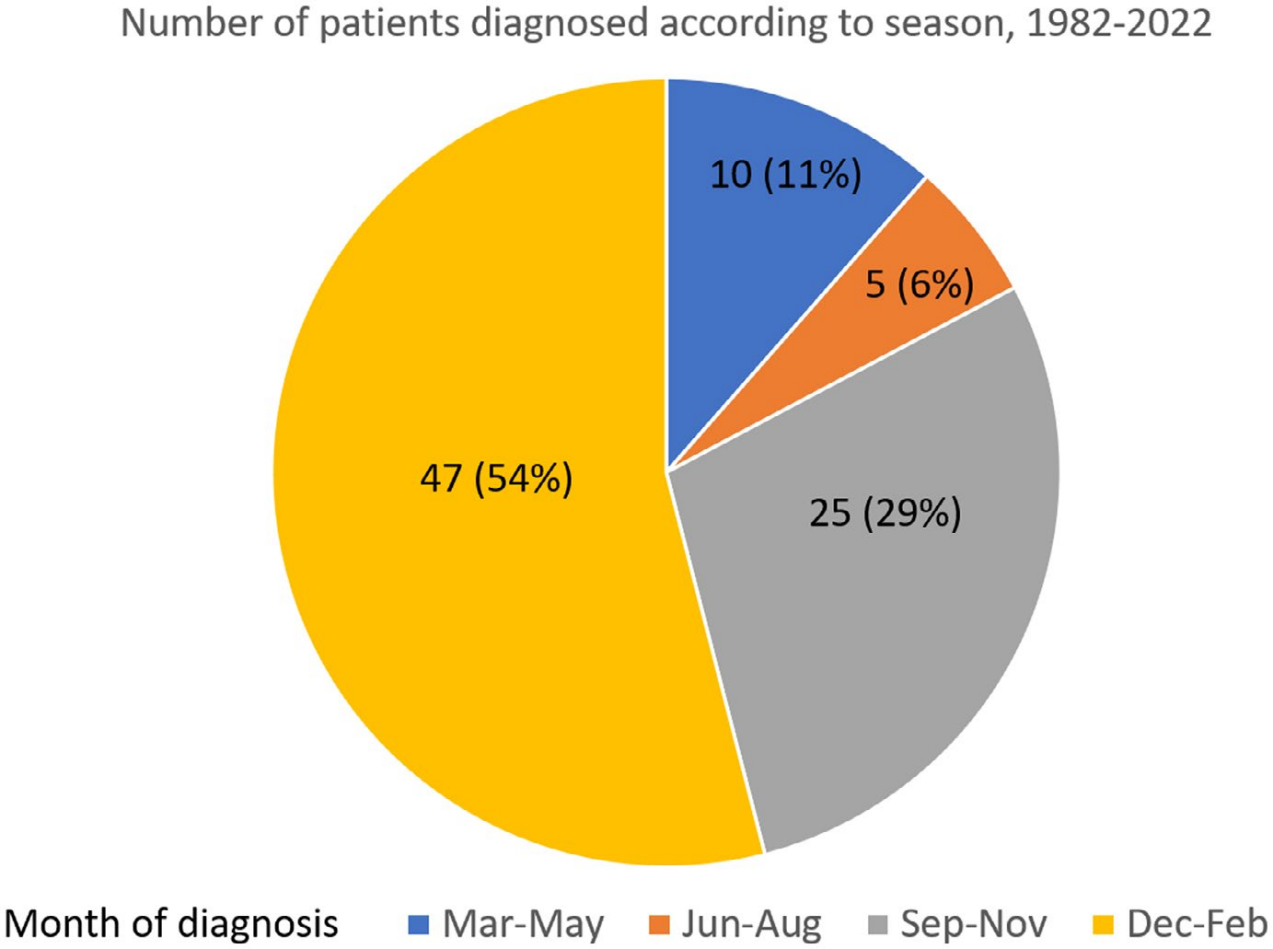
Month of diagnosis of all reported cases of BU.

The seasonal correlation remained prominent, with 21 out of the 27 recent cases (77.8%, 2017–2022) diagnosed during the autumn and winter months (September–February). Cumulatively, this accounts for 83% (72 out of 87 cases, 1982 onwards; Figure 3) of the total cases diagnosed in Japan. Further accumulation of cases is necessary to definitively establish any association between BU development and these factors.

## 4 DISCUSSION

### 4.1 Diagnosis of BU in Japan

In Japan, the NIID serves as the primary reference center for microbiological confirmation of BU cases. Samples, including cultures and skin biopsies, are directly sent from individual hospitals to the NIID. Given the long time needed for culturing *M*. *ulcerans*, which can take months, the preferred method of diagnosing BU is by PCR. By performing PCR, the IS*2404* insertion sequence specific to *M. ulcerans* is identified,^15^ and the following confirmation of the subspecies, *M. shinshuense*, is achieved through16s rRNA sequencing.^16^ *M. shinshuense* has been identified as the primary causative organism in the majority of the BU cases in Japan, with the first description dating back to 1989 in a report by Tsukamura et al.^17^ Subsequent genomic analyses suggest that *M. shinshuense* likely underwent intra‐species evolution, originating within Asia.^8^, ^16^, ^18^ Genomically, *M. shinshuense* possesses over 200 copies of the IS*2404* and lacks the serine/threonine protein kinase (STPK) gene of the pMUM001‐associated genes.^5^ It is known that *M. shinshuense* produces mycolactone A/B, in contrast to other subspecies.^19^ For example, the *M. ulcerans* strain found in Australia produces mycolactone C,^20^ while the ITM 98‐912 (MU98912) strain isolated in China that is very closely related to *M. shinshuense* is known to produce mycolactone D.^5^, ^8^

### 4.2 Treatment

In the most recent cohort of patients (2017–2022), 55.6% (15 out of 27 cases) were treated with a combination of rifampicin, clarithromycin, and levofloxacin.^21^ The pediatric cases opted to use tosufloxacin instead of levofloxacin, taking into account levofloxacin's safety profile in children. Additionally, moxifloxacin was used in some cases as their quinolone of choice, adopting a regimen that is commonly used in the Victoria region of Australia, where BU is prevalent.^22^

Surgical intervention was necessary in 51.9% of the cases between 2017 and 2022 (14 out of 27 cases), including procedures such as debridement and skin grafting, along with antimicrobial treatment. In one case, no antibiotics were used, and instead it was treated solely by surgical removal. The patient was a healthy 52‐year‐old, with a single small (3 cm) ulcer with no systemic symptoms. An initial biopsy raised suspicions of squamous cell carcinoma, prompting the surgical removal of the lesion. It was only after the excision that BU was diagnosed, and the patient has since remained free from recurrence. This represents a rare instance in which BU was unintentionally treated successfully with surgery alone. This success may be attributed to the patient's overall good health and the lesion's small size.

Historically, surgery held the position of first‐line treatment for BU.^23^ However, over the past two decades, the WHO recommended treatment for BU has been antibiotics. Surgical intervention is reserved for cases where the antimicrobial response is inadequate or in severe cases, after at least a 4‐week course of antibiotics.^6^ Therefore, although this specific case resulted in a favorable outcome, it should be interpreted as an exceptional case rather than a new treatment recommendation. Nonetheless, it still stands as a noteworthy case to report.

### 4.3 BU in Japan as a skin NTD

Typically, cases of BU are concentrated in sub‐Saharan African countries, where difficulties with geographic and financial access to medical care often result in delayed diagnosis and lack of treatment, which in turn leads to severe symptoms and poor prognosis. This context places BU in the group of skin NTDs. Initiatives have been introduced recently with the aim of addressing multiple skin NTDs together in co‐endemic regions.^24^, ^25^ One such measure to improve skin NTD control emphasizes the introduction of new technologies to expedite diagnosis and improve the management of dermatological conditions in remote and underserved areas, including BU.^26^

In contrast, Japan features a well‐established and accessible healthcare system. However, despite these advantages, BU cases in Japan still face diagnostic delays, leading to unnecessary disease progression and increased morbidity. A considerable contributing factor to these delays is the lack of awareness among clinicians regarding this disease. As highlighted in the skin NTD framework,^24^ raising awareness and education on BU is crucial to allow for earlier case detection and treatment. As one of the good practices that we carry out in Japan, we hold a 2‐day seminar annually focusing on skin infections, inviting approximately 100 young dermatologists in training nationwide. Training on BU is incorporated into the curriculum of non‐tuberculous mycobacterial skin infections, exemplifying a form of skin NTD integration.

BU is the most prevalent in sub‐Saharan Africa, where the climate is warm. While the mode of transmission of BU remains unclear, this apparent correlation between climate and BU prevalence demands attention. Interestingly, regions like South Asia or South America which share similar climates with Africa, report fewer BU cases. While factors such as the socio‐economic status and limited access to medical resources in rural Africa may contribute to higher transmission rates, heightened awareness of BU among patients and clinicians in Africa could be leading to more reporting of cases. The true magnitude of the disease burden in other regions is still obscure.

Japan is currently experiencing climate changes across all seasons due to global warming, prompting concerns about the potential emergence and spread of tropical infectious diseases in the country. Recent outbreaks of dengue fever have already underscored this concern, with mathematical predictions indicating its potential future endemicity in Japan.^27^ Similar predictions have been proposed for other infections, such as malaria and Zika,^28^ carried by mosquitos. While a definitive correlation between climate and transmission remains unclear, as mentioned above, there is a potential for BU to become a more widespread health problem in Japan in the near future. However, a contradiction to this correlation exists in Japan that many cases have been diagnosed during the winter months. One possible explanation could be the long incubation period of BU, with the initial infection occurring during the warmer months and becoming apparent in winter. Indeed, a study from Australia has suggested an incubation period of 4–5 months, which would align with the potential infection timeline in Japan, supporting this hypothesis.^29^

Nevertheless, further research into BU transmission is important to provide insights into the reasons why BU is so prevalent in tropical countries, and whether or not it has the potential to spread to other countries due to global warming. Although BU is not a ‘neglected’ disease in Japan in the traditional sense, it is a condition that requires much closer attention, akin to the focus given to affected tropical countries.

### 4.4 Global relevance of the Japanese cases

While we have highlighted the distinctive features of *M*. *shinshuense* infections in this report, concurrently, our cases hold considerable global relevance. Many characteristics are shared with BU cases from other countries, particularly in terms of clinical presentation and prognosis. No *M*. *ulcerans* strains have proven fatal, and the prognosis, when adequately treated, is good across all countries. In Japan, we have introduced some innovative methods for diagnosis and treatment modalities. Skin biopsy mapping has proven valuable in identifying the extent of necessary debridement, minimizing surgical excision.^30^, ^31^, ^32^, ^33^ Negative pressure wound therapy, now a mainstream option for wound treatment, was successfully used in one of our recent cases.^10^ These experiences may contribute to improving patient outcomes, in terms of achieving diagnostic speed and accuracy, and providing adequate treatment of BU cases. It is hoped that presenting these additional options would prove beneficial for other countries and offer insights into these new methods.

Phylogenetically, *M*. *shinshuense* represents a distinct subspecies that diverged from *M*. *marinum*, a common ancestor to the *M*. *ulcerans* strains, approximately 500 000 years ago.^34^ While closely related strains, such as the Ghanian subtype (Agy99)^34^ or MU98912,^5^, ^8^ share remarkably similar genomic structures with *M*. *shinshuense*, certain differences exist, such as the type of mycolactone produced, as discussed earlier. Exploring these genomic similarities may allow prediction of future behavior and enhance the understanding of BU transmission at both individual country and global levels. This is particularly important as the mode of BU transmission is not fully known,^2^ and its clarification could improve public health measures and preparation for any potential outbreaks.

A prior report of BU caused by *M. shinshuense* in China^35^ indicates that this subspecies is not confined to Japan. While it remains a single case, it has indicated the possibility of *M*. *shinshuense* being present in other Asian countries. Recently, a possible BU case from India was reported.^36^ The case was initially suspected histopathologically, and the remaining paraffin block sample was sent to NIID for PCR analysis. Unfortunately, the results were negative, and we were unable to confirm BU. However, this showcases the importance of maintaining vigilance for cases both in Asia and in other parts of the world where cases have not yet been identified or are not considered endemic. Disseminating information regarding BU caused by *M*. *ulcerans* and *M*. *shinshuense* remains important globally, irrespective of the current endemic status of various countries.

## 5 CONCLUSION

This article presents an updated epidemiological overview of BU cases in Japan, with a particular focus on discussing their similarities and differences compared to non‐Japanese cases. Despite a gradual increase in awareness and reported cases in Japan, there persists a lack of understanding among clinicians, and the undiagnosed ‘hidden population’ is believed to remain. The absence of a structured, government‐supported reporting system exacerbates this challenge. We anticipate a continued increase in BU cases in Japan, and there is a genuine risk that BU may become more endemic in Japan, as we have been observing in Australia. Through this article, we aim to renew interest in BU in Japan and elevate awareness of this important disease. Our goal is to generate high‐quality epidemiological data, contributing significantly to the global fight against BU as a skin NTD.

## FUNDING INFORMATION

This study was supported in part by grants from the Japan Agency for Medical Research and Development/Japan International Cooperation Agency (AMED) to Y.H. (JP20fk0108064, JP22jm0510004, JP22wm0225004, JP23wm0125007, JP23wm0225022), and from the Japan Society for the Promotion of Science (JSPS) for International Collaborative Research to Y.H. (JP69KK0217 and JP63KK0138) and for Scientific Research (C) to Y.H. (JP18K08312 and JP23K07665).

## CONFLICT OF INTEREST STATEMENT

The authors have no conflicts of interest to declare. Prof. Masashi Akiyama is an editorial board member of the *Journal of Dermatology* and a co‐author of this article. To minimize bias, he was excluded from all editorial decision‐making related to the acceptance of this article for publication.

## ETHICS STATEMENT

Written informed consent was obtained from patients for publication of this article. A copy of the written consent is available for review by the Editor‐in‐Chief of this journal on request.

## References

[1] World Health Organization . Ending the neglect to attain the sustainable development goals: a strategic framework for integrated control and management of skin‐related neglected tropical diseases. [cited 2023 Aug 1]. Available from: https://www.who.int/publications/i/item/9789240051423 2023. 10.1093/bjd/ljac031 36630642

[2] Yotsu RR , Suzuki K , Simmonds RE , Bedimo R , Ablordey A , Yeboah‐Manu D , et al. Buruli ulcer: a review of the current knowledge. Curr Trop Med Rep. 2018;5:247–256. 30460172 10.1007/s40475-018-0166-2 PMC6223704

[3] World Health Organization . Number of new reported cases of Buruli ulcer. [cited 2023 Aug 1]. Available from: https://www.who.int/data/gho/data/indicators/indicator‐details/GHO/number‐of‐new‐reported‐cases‐of‐buruli‐ulcer

[4] Mikoshiba H , Shindo Y , Matsumoto H , Mochizuki M , Tsukamura M . A case of typical mycobacteriosis due to *Mycobacterium ulcerans*‐like organism. Jpn J Dermatol. 1982;92:557–565. 6981005

[5] Nakanaga K , Hoshino Y , Yotsu R , Makino M , Ishii N . Nineteen cases of Buruli ulcer diagnosed in Japan from 1980 to 2010. J Clin Microbiol. 2011;49:3829–3836. 21880966 10.1128/JCM.00783-11 PMC3209082

[6] Yotsu R , Nakanaga K , Hoshino Y , Suzuki K , Ishii N . Buruli ulcer and current situation in Japan: a new emerging cutaneous mycobacterium infection. J Dermatol. 2012;39:587–593. 22486235 10.1111/j.1346-8138.2012.01543.x

[7] Yotsu R , Murase C , Sugawara M , Suzuki K , Nakanaga K , Ishii N , et al. Revisiting Buruli ulcer. J Dermatol. 2015;42:1033–1041. 26332541 10.1111/1346-8138.13049

[8] Suzuki K , Luo Y , Miyamoto Y , Murase C , Mikami‐Sugawara M , Yotsu RR , et al. Buruli ulcer in Japan. 2019. In: Pluschke G , Röltgen K , editors. Buruli ulcer: *Mycobacterium ulcerans* disease. Cham: Springer; 2019. 32091702

[9] Sato Y , Miura S , Watanabe D , Endo M , Miyamoto Y , Ishii N , et al. A case of Buruli ulcer in a child. Hifubyou Shinryou. 2018;40:1199–1202.

[10] Fujita S , Kawakami Y , Yamasaki H , Sugihara S , Miyake T , Miyamoto Y , et al. Coexistence of *Mycobacterium ulcerans* ssp. Shinshuense and *Mycobacterium avium* in a patient with Buruli ulcer‐compatible lesions. J Dermatol. 2020;47:e400–1. 32881049 10.1111/1346-8138.15542

[11] Fujimori T , Hagiya H , Iio K , Yamasaki O , Miyamoto Y , Hoshino Y , et al. Buruli ulcer caused by *Mycobacterium ulcerans* subsp. shinshuense: a case report. J Infect Chemother. 2023;29:523–526. 36813163 10.1016/j.jiac.2023.02.009

[12] Fukaura R , Koizumi H , Akashi N , Imai S , Murase C , Miyamoto Y , et al. Buruli ulcer with satellite lesions: a case report from Japan. J Dermatol. 2024. 10.1111/1346-8138.17274 39018184

[13] Omansen TF , Erbowor‐Becksen A , Yotsu R , van der Werf TS , Tiendrebeogo A , Grout L , et al. Global epidemiology of Buruli ulcer, 2010‐2017, and analysis of 2014 WHO programmatic targets. Emerg Infect Dis. 2019;25:2183–2190. 31742506 10.3201/eid2512.190427 PMC6874257

[14] Walker G , Friedman DN , O'Brien MP , Cooper C , McDonald A , Callan P , et al. Paediatric Buruli ulcer in Australia. J Paediatr Child Health. 2020;56:636–641. 31821679 10.1111/jpc.14704

[15] Herbinger KH , Adjei O , Awua‐Boateng NY , Nienhuis WA , Kunaa L , Siegmund V , et al. Comparative study of the sensitivity of different diagnostic methods for the laboratory diagnosis of Buruli ulcer disease. Clin Infect Dis. 2009;48:1055–1064. 19275499 10.1086/597398

[16] Nakanaga K , Ishii N , Suzuki K , Tanigawa K , Goto M , Okabe T , et al. "*mycobacterium ulcerans* subsp. shinshuense" isolated from a skin ulcer lesion: identification based on 16S rRNA gene sequencing. J Clin Microbiol. 2007;45:3840–3843. 17881555 10.1128/JCM.01041-07 PMC2168489

[17] Tsukamura M , Kaneda K , Imaeda T , Mikoshiba H . A taxonomic study on a mycobacterium which caused skin ulcer in a Japanese girl and resembled *Mycobacterium ulcerans* . Jpn J Tuberc Chest Dis. 1989;64:691–697. 2593460

[18] Käser M , Rondini S , Naegeli M , Stinear T , Portaels F , Certa U , et al. Evolution of two distinct phylogenetic lineages of the emerging human pathogen *Mycobacterium ulcerans* . BMC Evol Biol. 2007;27:177. 10.1186/1471-2148-7-177 PMC2098775 17900363

[19] Hong H , Spencer JB , Porter JL , Leadlay PF , Stinear T . A novel mycolactone from a clinical isolate of *Mycobacterium ulcerans* provides evidence for additional toxin heterogeneity as a result of specific changes in the modular polyketide synthase. Chembiochem. 2005;6:643–648. 15750996 10.1002/cbic.200400339

[20] Hong H , Demangel C , Pidot SJ , Leadlay PF , Stinear T . Mycolactones: immunosuppressive and cytotoxic polyketides produced by aquatic mycobacteria. Nat Prod Rep. 2008;25:447–454. 18497894 10.1039/b803101k PMC2730631

[21] Sugawara M , Ishii N , Nakanaga K , Suzuki K , Umebayashi Y , Makigami K , et al. Exploration of a standard treatment for Buruli ulcer through a comprehensive analysis of all cases diagnosed in Japan. J Dermatol. 2015;42:588–595. 25809502 10.1111/1346-8138.12851

[22] World Health Organization . Treatment of mycobacterium ulcerans disease (Buruli ulcer). Guidance for health workers 2012.

[23] Converse PJ , Nuermberger EL , Almeida DV , Grosset JH . Treating *Mycobacterium ulcerans* disease (Buruli ulcer): from surgery to antibiotics, is the pill mightier than the knife? Future Microbiol. 2011;6:1185–1198. 22004037 10.2217/fmb.11.101 PMC3243445

[24] Yotsu RR , Fuller LC , Murdoch ME , Revankar C , Barogui YT , Pemmaraju VRR , et al. World Health Organization strategic framework for integrated control and management of skin‐related neglected tropical diseases: what does this mean for dermatologists? Br J Dermatol. 2023;188:157–159. 36630642 10.1093/bjd/ljac031

[25] Yotsu RR , Fuller LC , Murdoch ME , van Brakel WH , Revankar C , Barogui MYT , et al. A global call for action to tackle skin‐related neglected tropical diseases (skin NTDs) through integration: an ambitious step change. PLoS Negl Trop Dis. 2023;17:e0011357. 37319139 10.1371/journal.pntd.0011357 PMC10270348

[26] Yotsu RR , Itoh S , Yao KA , Kouadio K , Ugai K , Koffi YD , et al. The early detection and case management of skin diseases with an mHealth app (eSkinHealth): protocol for a mixed methods pilot study in Côte D'ivoire. JMIR Res Protoc. 2022;11:e39867. 35922062 10.2196/39867 PMC9536527

[27] Hayashi K , Fujimoto M , Nishiura H . Quantifying the future risk of dengue under climate change in Japan. Front Public Health. 2022;10:959312. 35991044 10.3389/fpubh.2022.959312 PMC9389175

[28] Semenza JC , Rocklöv J , Ebi KL . Climate change and cascading risks from infectious disease. Infect Dis Ther. 2022;11:1371–1390. 35585385 10.1007/s40121-022-00647-3 PMC9334478

[29] Loftus MJ , Trubiano JA , Tay EL , Lavender CJ , Globan M , Fyfe JAM , et al. The incubation period of Buruli ulcer (*Mycobacterium ulcerans* infection) in Victoria, Australia—remains similar despite changing geographic distribution of disease. PLoS Negl Trop Dis. 2018;12:e0006323. 29554096 10.1371/journal.pntd.0006323 PMC5875870

[30] Kabuto M , Fujimoto N , Takahashi T , Nakanishi G , Nakanishi T , Tanaka T . Severe Buruli ulcer treated with minimal surgical excision after prior biopsy mapping. Acta Derm Venereol. 2016;96:982–984. 27026220 10.2340/00015555-2425

[31] Hayami T , Takahashi T , Kato T , Tanaka T , Fujimoto N . Mapping biopsy for Buruli ulcer self‐medicated with occlusive dressing. J Dermatol. 2018;45:72–75. 28891259 10.1111/1346-8138.14039

[32] Takahashi T , Kabuto M , Nakanishi G , Tanaka T , Fujimoto N . Histological and quantitative polymerase chain reaction‐based analysis of Buruli ulcer using mapping biopsy method. PLoS Negl Trop Dis. 2020;14:e0008051. 32569298 10.1371/journal.pntd.0008051 PMC7332088

[33] Takahashi T , Fujimoto N , Nakanishi G , Ishii N , Tanaka T . Mapping biopsy procedure on management of severe buruli ulcer due to *Mycobacterium ulcerans*, subspecies shinshuense. JAMA Dermatol. 2014;150:669–671. 24718693 10.1001/jamadermatol.2013.7497

[34] Qi W , Käser M , Röltgen K , Yeboah‐Manu D , Pluschke G . Genomic diversity and evolution of *Mycobacterium ulcerans* revealed by next‐generation sequencing. PLoS Pathog. 2009;5:e1000580. 19806175 10.1371/journal.ppat.1000580 PMC2736377

[35] Faber WR , Arias‐Bouda LM , Zeegelaar JE , Kolk AH , Fonteyne PA , Toonstra J , et al. First reported case of *Mycobacterium ulcerans* infection in a patient from China. Trans R Soc Trop Med Hyg. 2000;94:277–279. 10974998 10.1016/s0035-9203(00)90320-1

[36] Lahiri K , Dhar S , Saha A . Buruli ulcer a diagnostic challenge—a report from non‐endemic area. Indian J Dermatol. 2022;67:790–791. 36998861 10.4103/ijd.ijd_409_22 PMC10043679

